# The First National Program of Remote Cardiac Rehabilitation in Israel–Goal Achievements, Adherence, and Responsiveness in Older Adult Patients: Retrospective Analysis

**DOI:** 10.2196/36947

**Published:** 2022-11-16

**Authors:** Irene Nabutovsky, Daniel Breitner, Alexis Heller, Mickey Scheinowitz, Yarin Klempfner, Robert Klempfner

**Affiliations:** 1 Sackler Faculty of Medicine Tel Aviv University Tel-Aviv Israel; 2 Cardiac Prevention and Rehabilitation Institute Sheba Medical Center Ramat Gan Israel

**Keywords:** remote cardiac rehabilitation, mobile application, adherence, elderly patients, telehealth, telemedicine, cardiology, smartwatch, wearable, patient monitoring

## Abstract

**Background:**

Remote cardiac rehabilitation (RCR) after myocardial infarction is an innovative Israeli national program in the field of telecardiology. RCR is included in the Israeli health coverage for all citizens. It is generally accepted that telemedicine programs better apply to younger patients because it is thought that they are more technologically literate than are older patients. It has also previously been thought that older patients have difficulty using technology-based programs and attaining program goals.

**Objective:**

The objectives of this study were as follows: to study patterns of physical activity, goal achievement, and improvement in functional capacity among patients undergoing RCR over 65 years old compared to those of younger patients; and to identify predictors of better adherence with the RCR program.

**Methods:**

A retrospective study of patients post–myocardial infarction were enrolled in a 6-month RCR program. The activity of the patients was monitored using a smartwatch. The data were collected and analyzed by a special telemedicine platform. RCR program goals were as follows: 150 minutes of aerobic activity per week, 120 minutes of the activity in the target heart rate recommended by the exercise physiologist, and 8000 steps per day. Models were created to evaluate variables predicting adherence with the program.

**Results:**

Out of 306 patients, 80 were older adults (mean age 70 years, SD 3.4 years). At the end of the program, there was a significant improvement in the functional capacity of all patients (*P*=.002). Specifically, the older adult group improved from a mean 8.1 (SD 2.8) to 11.2 (SD 12.6). The metabolic equivalents of task (METs) and final MET results were similar among older and younger patients. During the entire program period, the older adult group showed better achievement of program goals compared to younger patients (*P*=.03). Additionally, we found that younger patient age is an independent predictor of early dropout from the program and completion of program goals (*P*=.045); younger patients were more likely to experience early program dropout and to complete fewer program goals.

**Conclusions:**

Older adult patients demonstrated better compliance and achievement of the goals of the remote rehabilitation program in comparison with younger patients. We found that older age is not a limitation but rather a predictor of better RCR program compliance and program goal achievement.

## Introduction

### Background

Cardiac rehabilitation (CR) is essential for comprehensive cardiac care, as it prevents future heart-related complications which often result in hospital readmissions and death [1,2]. Despite this strong evidence, patients often do not participate in traditional CR for several reasons, such as the location of the medical center, lack of transportation, and travel cost. Other factors include socioeconomic status, as well as behavioral and psychosocial reasons [3-5]. In contrast, remote CR (RCR) programs are individualized to each patient through telemedicine, regardless of where they are. This permits RCR to achieve all of the clinical goals set by CR while at the same time overcoming the many well-known barriers of CR. RCR has been shown to improve exercise adherence, increase physical activity level, and reduce the relative cost of treatment [6,7]. With this in mind, RCR has been introduced in Israel and is subsidized under its national health care coverage. As a result, Israel became one of the first countries where RCR began to play an important clinical role and is free of charge for all low risk patients with an indication for CR.

It is generally believed that older adults (>65 years old) struggle to use newer technologies. Various factors such as age-related cognitive impairment, vision or hearing difficulties, short-term memory loss, and physical limitations contribute to this assumption. Additionally, older adult patients have a preference for in-person communication with their physicians, resulting in a lower rate of acceptance of new technological applications [8,9]. Furthermore, a majority of older adult adults need assistance in using new digital devices, claiming they do not feel comfortable learning to use new technological devices such as smartphones or tablets on their own [9]. Some physicians are also less likely to send an older adult patient to a program that requires significant use of technology because they think that the patient is not likely to cope [10]. This is partly due to the current understanding that older adult patients have more difficulties absorbing new content and adapting to a changing environment, which poses another barrier to telemedicine [8] and digital health in general.

However, in recent years, there has been an understanding that older people are also willing and able to manage their health using the newest technologies [11]. Although the rates of mobile app usage among people aged 65 years or older is relatively small, holding steady with 20% usage [12], the introduction of telemedicine programs for older adults is increasing, ignoring the preconceived biases related to the ability of older adults in using technology [13]. These trends both emphasize the growing usage of technological devices by the older adult population and show the desire of older adult patients to control their health through digital devices.

It is well known that CR is essential for older adult patients due to this population having a higher risk of complications from cardiac-related causes compared to younger patients. However, there are contrasting results in this field of research. Previous studies have shown that older age is associated with a lower likelihood of participation in remote CR [14], while other studies have shown that patients over the age of 65 years are significantly more adherent to hospital-based CR [15]. However, the relationship between older adult patient adherence with remote CR has not been studied in detail.

Our goal is to further expand this area of research by comparing the adherence and program goals achievements between older and younger patients. The objectives of this study were as follows: to study patterns of physical activity, goal achievement, and improvement in functional level among patients over 65 years old undergoing RCR and compare them to those of younger patients; and to identify predictors of better adherence with the RCR program.

### RCR Program Description

The CR program is based on national guidelines provided by the Israeli Heart Society, specifically for comprehensive CR and the specific goals. A detailed description of the program and the Datos Health platform powering our RCR program was published previously [16]. In short, the main component of the program is structured exercise, monitored by a smartwatch capturing the essential data which are then transferred to a mobile app and presented to the patient and securely transferred to the medical operations center at our hospital (Multimedia Appendix 1 and 2). The remote care platform receives all the data generated by the smartwatch and the patient's mobile app and presents the information to the relevant care team member. The platform also includes care coordination tools scheduling follow-up remote visits with the multidisciplinary care team and provides easily accessible educational content that is pushed to the patients according to a prespecified plan. The platform allows the tracking of various measurements trends, interaction with the patient using asynchronous messaging and video chat, and collection of patients reported outcomes and questionnaires. The integrated information makes it possible to monitor, make decisions, and give recommendations regarding patient physical activity.

## Methods

### Study Cohort

Over the 18 months of the program's existence, we collected data on behavior patterns, training, and goal achievements from the first low-risk group of 306 patients rehabilitating under the RCR program at Sheba Medical Center in Israel. The participants of the group were both young and older adult patients. The collection of information and analysis were carried out retrospectively. The program goals were the same for all individuals regarding monthly exercise minutes (total exercise minutes and exercise in the target heart zone), and the exercise intensity was derived from the results of the exercise test reflecting the age-dependent maximal heart rate. Resistance training sessions and repetitions were similar but with individualized resistance. Basic characteristics, including a complete medical history, risk factors, and laboratory tests were collected. Training patterns were obtained by the smartwatch and then analyzed prospectively by the platform for 24 weeks. Improvement of an individual’s functional capacity was assessed as the change between the first (prerehabilitation) and the second (following 3 months of rehabilitation) exercise stress test (ergometry)–estimated metabolic equivalents of task (METs). Satisfaction with the program and the care received were assessed using a digital questionnaire.

### Study End Points

The primary end point of the study was to determine the difference in adherence to the RCR program goals among patients over 65 years of age compared with younger patients. The following variables were evaluated longitudinally in each month of the exercise program: the number of minutes of aerobic exercise (aerobic minutes), the number of aerobic minutes in the target heart rate, the assessment of perceived Borg scale, the number of daily steps, and the use of the RCR mobile app (number of weekly entries). Secondary end points included the improvement in functional capacity, the number of training sessions, and the satisfaction with the RCR program overall.

### Ethics Approval

All required ethics board approvals for this study have been given by the Sheba Medical Center committee (Sheba institutional review board approval #SMC-14-1553).

### Statistical Analysis

Descriptive statistics are presented according to variable characteristics and normality assumption evaluation. Baseline characteristics are presented as median, mean and SD, or percentages as appropriate. Group comparisons were performed according to data type and its respective distribution. A paired sample *t* test or Wilcoxon signed-rank test was used according to the data distribution to assess the differences between baseline and program completion values for the entire group and for age-stratified subgroups. A logistic regression model was constructed using the best subset method in order to determine independent predictors of selected program goals. The following covariables were introduced: age, sex, prerehabilitation METs, and indication for CR.

A *P* value <.05 was considered statistically significant. Tests were 2-sided. Statistical analyses were performed using R statistical software version 4.1.2 (The R Foundation for Statistical Computing).

## Results

The study included 306 patients, 26.1% (80/306) of whom were over 65 years old. Detailed characteristics of the group are summarized in Table 1. The main indications for CR were percutaneous coronary intervention (137/28, 148.8%) and myocardial infarction (138/281, 49.1%). Participants had a preserved or normal systolic function and no high-risk criteria, such as significant ischemia, angina, clinically significant ventricular arrhythmia, or signs of clinical instability. Older patients had significantly more individuals after coronary artery bypass graft (16/80, 21.6%) compared to younger patients (19/203,9.3%; *P*=.01). The median number of total minutes of aerobic training for 6 months was 183 minutes per week in the entire population. The older adult group achieved 222 minutes, whereas the younger group achieved 168 minutes (*P*=.003). Table 2 presents the total aerobic activity by program month. Additionally, the number of mobile app entries per week was significantly higher among older adult individuals during the entire duration of the program. Older adult patients had a median of 5.7 mobile app entries per week, whereas younger patients had 3.7 entries per week (*P*=.007).

Table 3 shows that the objective improvement in aerobic functional capacity after 3 months of RCR when compared to baseline was significant in the entire group (*P*=.001). Interestingly, prior to RCR initiation, there was a significant difference between the older adult group and the younger group in the baseline exercise capacity as expressed by METs (*P*=.002). However, this difference disappeared after 3 months of RCR.

Table 4 shows the percentage of those who achieved the main goals of RCR in the first 3 months of rehabilitation. These goals involved achieving 150 aerobic training minutes weekly and achieving 120 aerobic minutes in target heart rate per week. Among those who achieved these goals, the percentage of older adult patients was significantly higher when compared to younger patients (*P*=.03). The basic characteristics of patients who achieved the main goals versus those who did not during the third month of the program were also evaluated. Other than age, there was no significant difference between the groups of those who achieved versus those who did not achieve these goals. Older patients had significantly better completion rates of the three program goals: (1) completion of the full 3 months of RCR—the average age in the group of those who completed the program was 58.5 years while the average age of those in the group who dropped out was 55.5 years (*P*=.044); (2) achieving at least 600 aerobic minutes per month—the age of those who achieved this goal was 60 years while the average age of those who did not achieve this goal was 55 years (*P*=.001); (3) achieving at least 400 minutes per month of training in the target heart rate—the average age of those who achieved this goal was 63.7 years while the average age of those who did not achieve this goal was 56.9 years (*P*=.001).

**Table 1:** Baseline demographic and clinical characteristics of the study population.

Variables					Total population (N=306)			<65 years old (n=222)			>65 years old (n=80)			*P* value
Age (years), mean (SD)					57.59 (10.62)			53.01 (8.48)			70.16 (3.38)			<.001
Male sex, n (%)					229 (81.5)			171 (83.4)			56 (75.7)			.2
**Comorbidities, n (** **%)**														
	**Metabolic**													
		Dyslipidemia	85 (30.2)			57 (27.8)			27 (36.5)			.21		
		Hypertension	72 (25.6)			44 (21.5)			28 (37.8)			.009		
		Diabetes mellitus	17 (6)			11 (5.4)			6 (8.1)			.57		
	**Cardiovascular**													
		Myocardial infarction	138 (49.1)			101 (49.3)			35 (47.3)			.88		
		Atrial fibrillation	29 (10.3)			15 (7.3)			14 (18.9)			.01		
		Atrial flutter	4 (1.4)			3 (1.5)			1 (1.4)			>.99		
		Status post–coronary artery bypass graft	35 (12.5)			19 (9.3)			16 (21.6)			.01		
		Status post–percutaneous coronary intervention	137 (48.8))			103 (5.2)			33 (44.6)			.49		
**Physical and functional status**, **mean (SD)**														
	BMI (kg/m^2^)			27.76 (11.77)			28.15 (13.57)			26.91 (4.20)			.42	
	Systolic blood pressure (mmHg)			131.18 (20.13)			128.35 (19.78)			139.79 (18.74)			.002	
	Diastolic blood pressure (mmHg)			75.71 (12.12)			75.21 (12.18)			77.46 (11.90)			.32	
	Pre–heart rate at maximum effort			140.01 (19.66)			144.98 (18.11)			125.99 (17.09)			<.001	
	Post–heart rate at maximum effort			145.35 (18.87)			148.77 (18.44)			136.24 (17.09)			<.001	
	Pre-METs^a^ (kcal/kg/min)			9.49 (2.88)			9.98 (2.76)			8.11 (2.80)			<.001	
	Post-METs (kcal/kg/min)			11.38 (7.00)			11.42 (3.13)			11.25 (12.62)			.88	

^a^MET: metabolic equivalent task.

**Table 2:** Total aerobic minutes per month.

Month	<65 years old (min), median (n=222)	>65 years old (min), median (n=80)	*P* value
1	167	215	.002
2	166	230	.001
3	158	212	.002
4	150	213	.002
5	165	213	.004
6	142	168	.004

**Table 3:** Exercise capacity before and after RCR.

Max METs^a^	<65 years old (n=222)	>65 years old (n=80)	*P* value
Pre-RCR^b^	9.98	8.11	.001
Post-RCR	11.42	11.25	.33

^a^MET: metabolic equivalent of task

^b^RCR: remote cardiac rehabilitation.

**Table 4:** RCR outcomes by age group.

RCR^a^ outcomes	<65 years old, n (%) (n=222)	>65 years old, n (%) (n=80)	*P* value	
Reached target heart rate minutes 1st month	31 (14.1)	24 (29.8)	.04	
Reached target heartrate minutes 2nd month	38 (17.3)	31 (38.6)	.01	
Reached target heart rate minutes 3rd month	102 (45.8)	42 (52.9)	.21	
Reached total aerobic minutes 1st month	150 (67.6)	66 (82.5)	.04	
Reached total aerobic minutes 2nd month	147 (66.2)	69 (86.2)	.03	
Reached total aerobic minutes 3rd month	141 (63.4)	67 (84.3)	.02	

^a^RCR: remote cardiac rehabilitation.

### Independent Predictors of Goal Completion

A logistic regression model was constructed to predict each of the 3 main program goals. Higher age was consistently an independent predictor of achieving the RCR aerobic exercise goals of completing at least 600 aerobic minutes per month (odds ratio 1.07, 95% CI 1.03-1.13; *P*=.007) and completing at least 400 minutes per month of training in the target heart rate (odds ratio 1.09, 95% CI 1.03-1.15; *P*=.008).

There was no significant difference between older adult and younger patients in the number of daily steps or in the amount of weekly use of the mobile app. However, a significant difference was observed in the number of aerobic workouts per week: the median number of workouts per week in the older adult group was 6.7 versus 3.7 in the younger group (*P*=.002).

Over 85.9% (263/306) of patients reported feeling safe and satisfied with RCR, and 83.9% (257/306) of patients answered that the program helped them maintain a healthy lifestyle.

## Discussion

### Principal Findings

The principal findings of our study are the following: participants of the RCR adhered to the program and most attained the prespecified goals, older adult patients had higher compliance and were more likely to reach RCR goals compared to younger participants, and older adult patients had a significant absolute improvement in functional capacity assessed objectively by the stress test.

### Comparison With Prior Work

Previous studies have mentioned factors such as preexisting health conditions and lower physical functioning as additional barriers which make older adult patients unable to benefit from CR compared to younger patients [17]. However, our results did not find that these factors were significant barriers to older adult patients’ remote CR adherence. Our study demonstrated that older adult patients were able to effectively adhere to and use modern technology during the program. Our results found that older adult patients had greater program compliance than that originally thought. This could be due to several factors. First, patients with previous cardiovascular events (ie, acute coronary syndrome or revascularization procedures) who are older are usually at a higher risk compared to younger patients [18]. Their higher risk status could have motivated them to participate more actively when compared to younger patients. Other studies have also shown that older adult patients seem to be more attentive to their health conditions, whereas younger patients might be less attentive because they often consider themselves to have a strong recovery ability [19]. Second, other studies have shown that higher risk patients in CR participate in more CR sessions than do lower risk patients [15]. Although we did not stratify patients into these same categories, we showed that older adult patients attended more remote CR sessions than did their younger counterparts. This was found to lead to better program goal achievements. One explanation for this could be that older adult patients are generally retired and have more free time compared to younger patients. This concept has been previously studied, showing that employment status can be a negative predictor of CR adherence, as older patients tend to be retired and have more free time for training [20]. Program goal achievement was correlated with the significant improvement in functional capacity where the older adult group reached similar levels of exercise capacity (assessed in METs) as did young patients despite the difference in functional capacity at the beginning of the program. Improvements in performance have been shown to be associated with improved survival and overall well-being [21,22].

A common misperception is that older adult people (>65 years old) are hesitant to accept new technologies. Several studies state barriers such as lack of knowledge or fear of misusing remote CR technology [23]. However, other studies also report that older adult patients were eager to adopt new technologies and had no difficulty using remote CR devices [23,24]. The second group of studies above aligns well with the findings of our study. Older adult patients were effectively able to use remote CR technology. Moreover, we found that these older adult patients were more consistent in achieving the goals of the program when compared with younger patients.

### Strengths and Limitations

The strength of this study is that it is the first and exclusive study of a new national telerehabilitation program fully subsidized by the Ministry of Health. Moreover, the analysis carried out in this study covered a relatively large cohort of patients and carefully analyzed multiple aspects of their performance over a 6-month period. Nevertheless, our study has a number of limitations. First, it used a retrospective design and included a relatively low risk population, with most of the participants being men. This is unsurprising, as secondary prevention treatments are underused in women with coronary heart disease [25]. Second, we present the experience of a single center following a specific RCR protocol using a dedicated digital health platform. At the time of the study, there were no other cardiology centers in our country offering a similar program to patients, so it was impossible to create a multicenter study. In the future, it is essential to collect data from multiple sites to increase the generalizability of the results and to allow for the comparison among different programs.

### Future Directions

Multicenter prospective research is necessary in order to assess the generalizability of these findings. Furthermore, now having an understanding of the successful implementation of the program even among low-tech older adult people, we further seek to expand the implementation of telerehabilitation usage among patients at medium and high risk, for example, patients with heart failure. The recent experience of the COVID-19 pandemic has further emphasized the importance of implementing telecare for all types of patients without exception.

### Conclusions

Our study showed that older adult patients demonstrated better compliance with the remote CR program in most aspects. Higher age was an independent predictor of better compliance with program goals. Given these results, we suggest that CR programs are more suitable for older adult patients than initially thought. However, due to the misconceptions about their ability to use technology, older adult patients remain underrepresented in current remote digital health studies. Future studies need to be conducted to understand this relationship and explore the potential benefit of remote rehabilitation in other fields of medicine among older adult patients.

## Appendix

Multimedia Appendix 1 Care management screens of the remote cardiac rehabilitation.

Multimedia Appendix 2 The remote cardiac rehabilitation patient mobile app (iOS and Android).

## Abbreviations

CR: cardiac rehabilitation
MET: metabolic equivalent of task
RCR: remote cardiac rehabilitation

## Data Availability

The data set used for this study contains a great number of details per patient each and cannot, per institutional review board approval, be shared even in anonymized form. Requests for partial anonymized data for specific projects can be discussed with the corresponding author (RK).
