# Supplementary figures and images for: The First National Program of Remote Cardiac Rehabilitation in Israel–Goal Achievements, Adherence, and Responsiveness in Older Adult Patients: Retrospective Analysis
Source: JMIR Cardio. 2022 Nov 16;6(2):e36947. doi: 10.2196/36947 (PMC9713616; doi:10.2196/36947)

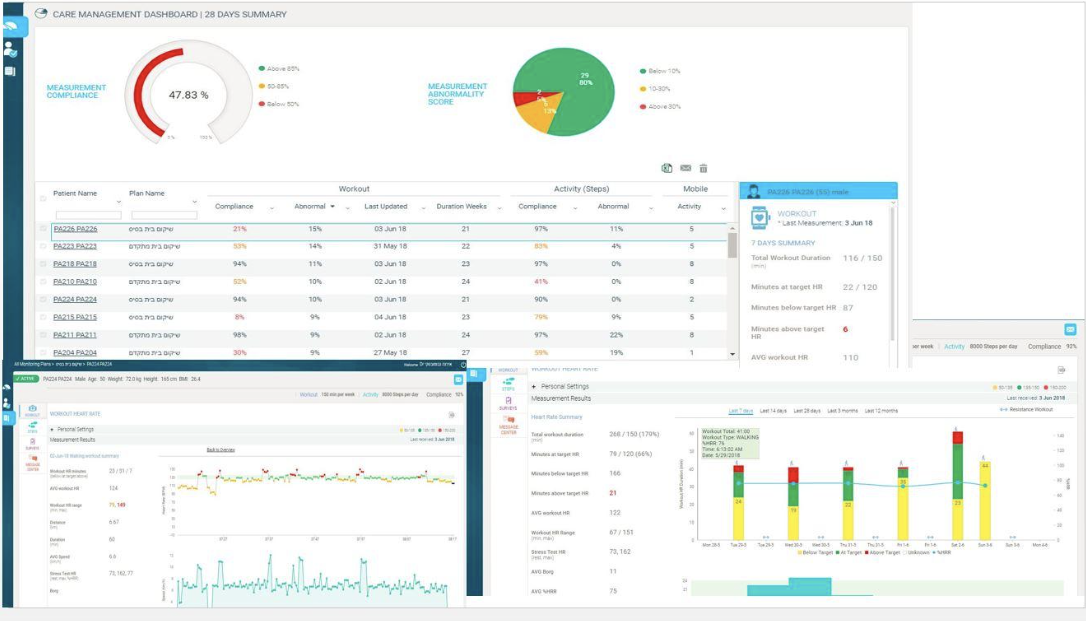

Supplement: Multimedia Appendix 1 [file cardio_v6i2e36947_app1.png]

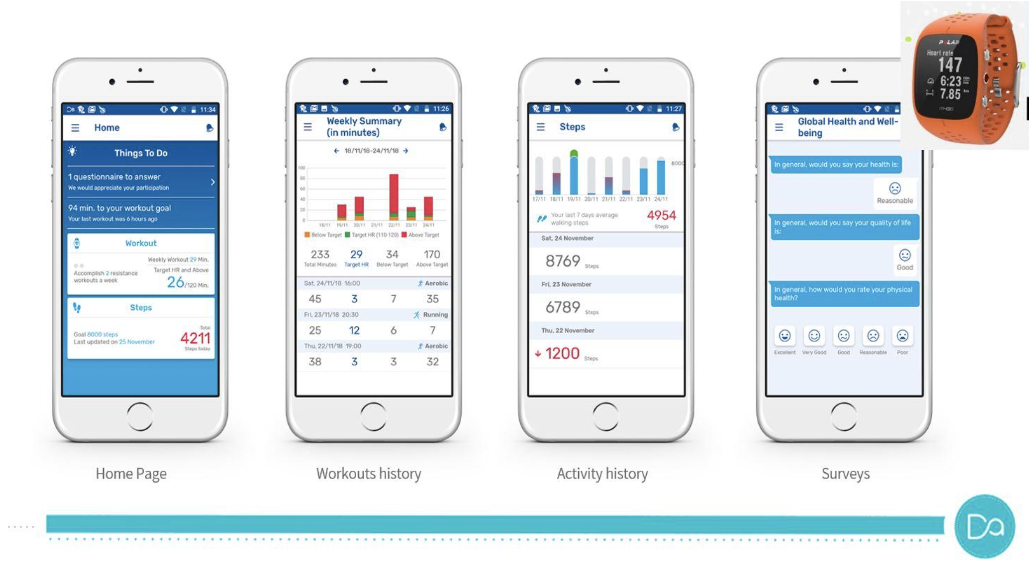

Supplement: Multimedia Appendix 2 [file cardio_v6i2e36947_app2.png]
